# Supplementary material for: Scavenger receptor B1 facilitates the endocytosis of Escherichia coli via TLR4 signaling in mammary gland infection
Source: Cell Commun Signal. 2023 Jan 5;21:3. doi: 10.1186/s12964-022-01014-y (PMC9813905; doi:10.1186/s12964-022-01014-y)
Supplement: Supplementary file 2 — Additional file 1. The figures represent morphological and proliferative characteristics, representative metaphase distribution, lumen structures in GMECs, EGFPC1 transfected GMECs, quantitative analysis results of MTT cytotoxicity assay, sequence of control siRNA-EGFP and esiRNA used for downregulation of SCARB1 and table with primer details. [file 12964_2022_1014_MOESM2_ESM.doc]

**Supplementary 1**

**Fig. 1 Morphological and proliferative characteristics of GMECs. a,** Growth curve of GMECs from different passages show a gradual increase in growth represented by sigmoidal curve over a span of 10 days. **b,** Quantitative analysis results of MTT assay shows linearity in the rate of proliferation for early (P2) and late passage cells (P13, P14 and P15) over the span of 7 day at 570nm. **c,** Giemsa stained GMECs at early and late passages (P2, P3, P4, P13, P14 and P15) show prominent nuclear and/or cytoplasmic morphology typical of epithelial cells (×10).

**Fig. 2** Representative Metaphase Distribution and Karyotype of GMECs showing 30 pair of chromosomes specific to Goat (*Capra hircus*).

**Fig. 3** Lumen like structures observed in GMECs after induction in P2 and P13 visualized under ×40 and ×4 magnification respectively.

**Fig. 4 GMECs transfected with EGFPC1.** **a,** GMECs transfected by EGFPC1 plasmid 24 h after transfection with Turbofect under Inverted fluorescent Microscope (×100). **b,** Vector map of EGFPC1 plasmid.

**Fig. 5** Quantitative analysis results of MTT cytotoxicity assay shows decrease in the rate of percentage cells viability of GMECs with increase in MOIs at time points (3 h, 6 h and 24 h). Minimum cytotoxicity is observed at early MOIs and highest at MOI 1000 at all time points.

**Table 1: Sequence of control siRNA-EGFP as well as esiRNA used for downregulation of SCARB1**

| **esiRNA** | **Sequence** | **Transfection reagent** |
| --- | --- | --- |
| esiRNA-SCRAB1 | GGAGTCCGGAATCCAAAACCTCAGCACCTGCAGGTTCAATGCACCCTTGTTTCTCTCCCATCCTCACTTCTACAATGCTGACCCAGTCCTGGCAGAGGCAGTGACTGGCCTCCATCCTCCATCCTAACCCAAAAGAACATTCCTTGTTCTTGGACATCCACCCGGTCACTGGAATCCCCATGAACTGCTCTGTGAAACTGCAGCTGAGCCTCTTCCTTAAGTCTGTGAAAGGCATCGGACAAACTGGGAACATCCAGCCAGTG | X-tremeGENE 360 Transfection Reagent |
| esiRNA-EGFP | GTGAGCAAGGGCGAGGAGCTGTTCACCGGGGTGGTGCCCATCCTGGTCGAGCTGGACGGCGACGTAAACGGCCACAAGTTCAGCGTGTCCGGCGAGGGCGAGGGCGATGCCACCTACGGCAAGCTGACCCTGAAGTTCATCTGCACCACCGGCAAGCTGCCCGTGCCCTGGCCCACCCTCGTGACCACCCTGACCTACGGCGTGCAGTGCTTCAGCCGCTACCCCGACCACATGAAGCAGCACGACTTCTTCAAGTCCGCCATGCCCGAAGGCTACGTCCAGGAGCGCACCATCTTCTTCAAGGACGACGGCAACTACAAGACCCGCGCCGAGGTGAAGTTCGAGGGCGACACCCTGGTGAACCGCATCGAGCTGAAGGGCATCGACTTCAAGGAGGACGGCAACATCCTGGGGCACAAGCTGGAGTACAACTACAACAGCCACAACGTCTATATCATGGCCGACAAGCAGAAGAACGGCATCAAGGTGAACTTCAAGATCCGCCACAACATCGAGGACGGCAGCGTGCAGCTCGCCGACCACTACCAGCAGAACACCCCCATCGGCGACGGCCCCGTGCTGCTGCCCGACAACCACTACCTGAGCACCCAGTCCGCCCTGAGCAAAGACCCCAACGAGAAGCGCGATCACATGGTCCTGCTGGAGTTCGTGACCGCCGCCGGGATCACTCTCGGCATGGACGAGCTGTA | X-tremeGENE 360 Transfection Reagent |

**Table 2: Primer details for amplification of genes.**

| **Nature of Primer** | **Primer sequence** | | **Size of Amplicon** |
| --- | --- | --- | --- |
| *β-Actin* | Forward primer | 5'-CTCTTCCAGCCTTCCTTCCT-3' | 100 bp |
|  | Reverse primer | 5'-TAAAGGTCCTTGCGGATGTC-3' |  |
| *GAPDH* | Forward primer | 5'-GCAAGTTCCACGGCACAG-3' | 249 bp |
|  | Reverse primer | 5'-GGTTCACGCCCATCACAA-3' |  |
| *CSN-2* | Forward primer | 5'-ATGAAACTTCTCATCCTTACCTGTCTT-3' | 206bp |
|  | Reverse primer | 5'-CCAATATCCTTGCTCAGTTCATT-3' |  |
| *SCARB1* | Forward primer | 5'-TCCTACTGCTCATCCCCATC-3' | 185bp |
|  | Reverse primer | 5'- GGCATCTTTGGAACCCTACA -3' |  |
| *TLR4* | Forward primer | 5'-AGATGGCAACACTTAGAA-3' | 141bp |
|  | Reverse primer | 5'-GATGCTGCGGGCCCGCAA-3' |  |
| *TRIF* | Forward primer | 5'-GCACGTCTAGCCTGCTTAC-3' | 106bp |
|  | Reverse primer | 5'-TTGCGGGCCCGCAGCATCT-3' |  |
| *MyD88* | Forward primer | 5'-CGGATGGTGGTGGTTGTCT-3' | 142bp |
|  | Reverse primer | 5'-GGAACTCTTTCTTCATTGGCTTGT-3' |  |
| *IRF3* | Forward primer | 5'-TTGTGAACTCAGGGGTCAGG-3' | 125bp |
|  | Reverse primer | 5'-TGGGCTCAAGTCCATGTCAC-3' |  |
| *TRAF3* | Forward primer | 5'-TAACTGCTGCATTCGCTCCA-3' | 100bp |
|  | Reverse primer | 5'-GGAACACAAAGCTGGGGTTG-3' |  |
| *TRAF6* | Forward primer | 5'-CGGTGACTCTCTCCAGCTC-3' | 194bp |
|  | Reverse primer | 5'-TGGACATTTGTGACCTGCAT-3' |  |
| *MAPK1* | Forward primer | 5'-GCAACGACCACATCTGCTAC-3' | 100bp |
|  | Reverse primer | 5'-AGGTTGGAAGGCTTGAGGTC-3' |  |
| *TNF-α* | Forward primer | 5'-TGGTTCAGACACTCAGGT-3' | 75bp |
|  | Reverse primer | 5'-CGCTGATGTTGGCTACAA-3' |  |
| *NF-kB* | Forward primer | 5'-CAGCTCACAGATCGGGAAAAG-3' | 115bp |
|  | Reverse primer | 5'-CGGTGCTGTCTGGAAGGAA-3' |  |
| *INF-β* | Forward primer | 5'-TGCCAGAACCTCCTGTG-3' | 305bp |
|  | Reverse primer | 5'-TCGGTCGTGTCTCCCAT-3' |  |
| *IL-8* | Forward primer | 5'-TGAGAGTGGGCCACACT-3' | 103bp |
|  | Reverse primer | 5'-CACAACCTTCTGCACCCACTT-3' |  |
