# Supplementary material for: Scavenger receptor B1 facilitates the endocytosis of Escherichia coli via TLR4 signaling in mammary gland infection
Source: Cell Commun Signal. 2023 Jan 5;21:3. doi: 10.1186/s12964-022-01014-y (PMC9813905; doi:10.1186/s12964-022-01014-y)
Supplement: Supplementary file 3 — Additional file 2. The figures represent biochemical tests performed for bacterial identification, E.coli induced SCARB1 expression in GMECs, Phenotypic analysis of GMECs transfected with NC esiRNA-EGFP, graphs showing time dependent knockdown efficiencies on silencing of SCARB1, Protein-Protein Interaction network and Toll-like receptor signaling pathway, SCARB1 protein structures, Tables showing KEGG, GO and Reactome enriched pathways. [file 12964_2022_1014_MOESM3_ESM.doc]

**Supplementary 2**

**Fig. 1** **Screening of Goats with mastitis/mammary gland infectionsa,** Pink colonies of *E.coli* on MacConkey agar, **b,** *E.coli* showing metallic sheen on EMB agar, **c,** Gram stain for identification of *E. coli* from clinical mastitis, **d,** Positive IMViC tests for mastitic milk.

**Fig. 2 *E.coli* induced SCARB1 expression in GMECs. a,** No significant expression of SCARB1 is observed at 3 h post infection at mRNA and protein levels with increasing MOIs (1:100, 1:300, 1:300). **b,** Decreased expression of SCARB1 mRNA and protein at 24 h post infection with increasing MOI is detected. **c,** Cells treated with LPS show increased significant expression of SCARB1 with increasing LPS concentration (1, 10 and 50 μg/ml) for 6 h. Quantitative PCR data were normalized to GAPDH and β-Actin. All data are presented as the mean ± SEM from three experiments. **P* < 0.05 and not significant (NS).

**Fig. 3 Silencing of SCARB1 a,** Phenotypic analysis of GMECs transfected with NC esiRNA- EGFP (negative control). **b,** The time-dependent knockdown efficiencies on SCARB1 mRNA levels detected at 24, 48, and 72 h post transfection with NC-esiRNA and esi-SCARB1. SCARB1 is efficiently silences at 72 h post transfection. **c,** SCARB1 mRNA expression at 72 h post transfection at MOIs (1:100, 1:300 and 1:500). MOI 1:500 is the ideal MOI where the SCARB1 expression is increased after 72 h post silencing. Quantitative PCR data were normalized to GAPDH and β-Actin. The values are the mean ± SEM for triplicate samples. All data are presented as the mean ± SEM from three experiments. **P* < 0.05 and not significant (NS).


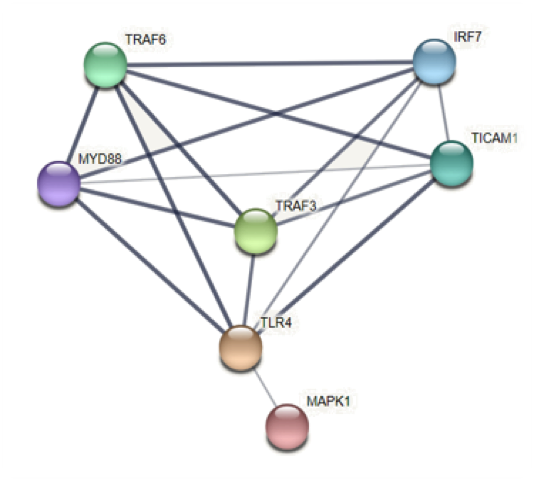


**Fig. 4** PPI network suggests interactions of TLR4 pathway genes with Scavenger class B receptor proteins on and the SIGNOR database suggests that Papain-like proteinase and NF-kB signaling involved in the process.

**Fig. 5 Schematics of Toll-like receptor signaling pathway. TLR signaling highlighted in purple edges. The enriched genes are shown in pink colour.**

**Fig. 6 a,** SCARB1 superimposed structures built using Homology Modeling. RAMACHANDRAN Plot shows 88.1% of residues are in core region, 11% are in allowed region and 0.6% is in disallowed region. **b,** Structure predicted using Ab initio method – Rosetta Program. RAMACHARDRAN Plot shows 88.3% residues are in allowed region and 11.7% residues are in allowed region, none of the residue is in dis-allowed region.

**Table 3: Enri**ched pathways from KEGG database.

| **Pathways** | **FDR Adjusted P-Value** | ***Genes* involved** |
| --- | --- | --- |
| Toll-like receptor signaling pathway | 1.63E-12 | MAPK1, MYD88, NFKB1, TICAM1, TLR4, TRAF3, TRAF6, CXCL8 |
| MAPK signaling pathway | 0.021286 | MAPK1, MYD88, NFKB1, TRAF6 |

**Table 4: Enriched processes from Reactome database.**

| **Processes** | **ID** | **FDR Adjusted P-Value** | ***Genes* involved** |
| --- | --- | --- | --- |
| Toll-like Receptor Cascades | REAC:R-GGA-168898 | 2.71E-08 | MYD88, NFKB1, TICAM1, TLR4, TRAF3, TRAF6 |
| TRAF6 mediated induction of NF-κB and MAP kinases upon TLR7/8 or 9 activation | REAC:R-GGA-975138 | 2.82E-07 | MYD88, NFKB1, TICAM1, TLR4, TRAF6 |
| Toll-like receptors (TLR) cascades | REAC:R-GGA-437987 | 4.45E-05 | MYD88, NFKB1, TRAF6 |

**Table 5: Enriched terms from GO analysis (Biological process).**

| **GO Terms** | **ID** | **FDR Adjusted P-Value** | ***Genes* involved** |
| --- | --- | --- | --- |
| Cellular response to molecule of bacterial origin | GO:0071219 | 3.05E-09 | MAPK1, MYD88, NFKB1, TLR4, TRAF6, CXCL8 |
| Biological process involved in interspecies interaction between organisms | GO:0044419 | 9.36E-09 | MAPK1, MYD88, NFKB1, SCARB1, TICAM1, TLR4, TRAF3, TRAF6, CXCL8 |
| Immune response | GO:0006955 | 0.000167 | MAPK1, MYD88, TICAM1, TLR4, TRAF3, TRAF6, CXCL8 |
| Regulation of defense response | GO:0031347 | 0.00046 | MYD88, NFKB1, TLR4, TRAF3, CXCL8 |
| Interleukin-12 production | GO:0032615 | 0.000775 | NFKB1, TLR4, TRAF6 |
| Positive regulation of MAPK cascade | GO:0043410 | 0.000804 | MAPK1, MYD88, TLR4, TRAF3, TRAF6 |
| Regulation of I-kappaB kinase/NF-kappaB signaling | GO:0043122 | 0.000951 | MYD88, TLR4, TRAF3, TRAF6 |
| Defense response | GO:0006952 | 0.002661 | MYD88, NFKB1, TICAM1, TLR4, TRAF3, CXCL8 |
| Toll signaling pathway | GO:0008063 | 0.003145 | MYD88, TRAF3 |
| NIK/NF-kappaB signaling | GO:0038061 | 0.013442 | MYD88, TLR4, TRAF6 |
