# Supplementary material for: Scavenger receptor B1 facilitates the endocytosis of Escherichia coli via TLR4 signaling in mammary gland infection
Source: Cell Commun Signal. 2023 Jan 5;21:3. doi: 10.1186/s12964-022-01014-y (PMC9813905; doi:10.1186/s12964-022-01014-y)
Supplement: Supplementary file 4 — Additional file 3. Histopathology images representing expression of normal and mastitic goat mammary gland tissue. [file 12964_2022_1014_MOESM4_ESM.doc]

**Supplementary 3**

**Histopathology of goat mammary gland tissue,** **a** Weak expression of SCARBI in healthy goat mammary gland tissue. **b** Strong expression of SCARBI in mastitic goat mammary gland tissue. (Scale 50 μm).
